# Violent relationships at the social-ecological level: A multi-mediation model to predict adolescent victimization by peers, bullying and depression in early and late adolescence

**DOI:** 10.1371/journal.pone.0174139

**Published:** 2017-03-30

**Authors:** Xavier Oriol, Rafael Miranda, Alberto Amutio, Hedy C. Acosta, Michelle C. Mendoza, Javier Torres-Vallejos

**Affiliations:** 1 Facultad de Educación, Universidad Andres Bello, Santiago de Chile, Chile; 2 Department of Psychology, Universidad Continental, Huancayo, Perú; 3 Department of Social Psychology and Methodology of the Behavioral Sciences, Faculty of Psychology, Donostia-San Sebastian, Spain; 4 Escuela de Psicología, Universidad Adolfo Ibáñez, Santiago de Chile, Chile; 5 Facultad de Ciencias Sociales y Humanidades, Universidad Autónoma de Chile, Santiago de Chile, Chile; 6 Pontificia Universidad Católica de Valparaíso Valparaiso, Chile; University of Westminster, UNITED KINGDOM

## Abstract

**Background:**

From the social-ecological perspective, exposure to violence at the different developmental levels is fundamental to explain the dynamics of violence and victimization in educational centers. The following study aims at analyzing how these relationships are produced in the Peruvian context, where structural violence situations exist.

**Methods:**

A multi-mediation structural model with 21,416 Peruvian adolescents (*M =* 13.69; *SD =* 0.71) was conducted to determine the influence of violence in the school environment on violence perceived within school and violence exercised by teachers. In addition, it was also intended to determine whether these violent relationships predict depression through loneliness, and bullying through peer victimization. The existence of differences between early and late adolescence was also verified.

**Results:**

Results confirm that violence in the school setting has high influence on violence exercised by adolescents and teachers within the school. Teacher violence is the most important predictor of depression through loneliness, and encourages peer victimization and the emergence of aggressive behavior. Exposure to violence exercised by support sources—teachers and classmates—explains more than 90% of the total variance explained in bullying behavior. Differences were found between early and late adolescence models.

**Conclusion:**

The high prevalence of structural violence in school settings facilitates the bullying/victimization dynamics within school. From a social-ecological perspective, this result suggests the importance of network cooperation at a mesosystem level, with teachers from educational centers playing a crucial role in the prevention of bullying/victimization.

## Introduction

From the social-ecological perspective [1], relationships between developmental environments and the dynamics that occur within them are fundamental to understand how the risk of violence, its protective factors, and depression in adolescence are constructed (for revision, [2]). Thus, a central tenet of this theory is that individual development is influenced by the ongoing qualities of the child’s social settings and the interactions between these settings (e.g., family, peers, schools, communities) [3]. An educational model oriented towards a conception of the ecological development of the individual cannot help but consider the varied forms of interaction and interchange that children experience both inside and outside schools [4, 5, 6].

In the social-ecological model, the mesosystem refers to the relationships between the different microsystems. This concept is of paramount importance for studies on bullying [7, 8, 9] and, thus, for this specific study. Specifically, student-teacher relationships are microsystems that consist of the multiple interrelated perceptions that both parties have about their interactions [10, 11]. Perceptions are important because they are real, from a psychological standpoint, and they have the power to influence the behavior of each party significantly [12].

During adolescence, the school environment serves a pivotal role in the development process and asserts itself as one of the most important socialization spaces [13]. In addition, affective relationships are established with peers and adults like teachers, who acquire special significance in this developmental stage [14, 15]. School, as a socializing setting, forms part of a neighborhood or geographical zone which, in turn, influences the experiences and everyday relationships at school [16,11]. Therefore, the study of the structural violence present in zones or neighborhoods where schools are located has become a growing interest over the last years, as it may help explaining bullying phenomena [17, 2, 16]. From the ecological perspective, understanding the influence of diverse factors on victimization/bullying dynamics as well as the relationships between the different actors becomes necessary for adopting effective preventive measures. The main factors to be studied are:

1) Contextual factors: According to the *Theory of Structural Violence* developed by Galtung [18], the extreme adversity conditions to which individuals are subjected within a society may be considered structural violence conditions when they are embedded in the social, political and economic organization of society. Peru is one of the countries where the *Shining Path terrorist activity* from 1980 provoked structural consequences, which makes it be regarded as a country ravaged by violence until today [19]. During decades, many of the population was frequently exposed to stress situations that generated important consequences such as depression, anxiety and post-traumatic stress disorders (PTSD) [20]. Currently, violence is considered a pervasive issue in Peru, which manifests in all contexts, such as workplaces, streets, public spaces, and affects equally men, women, adolescents and children [21]. Among Peruvian youngsters, these environments affected by structural violence translate into participation in street gangs and high consumption of alcohol from early ages. Furthermore, 50% of regular consumers start consuming alcohol at age 13, while 90% starts before 16, and this is encompassed by a high prevalence of violence in schools [22, 23]. Schools located in settings where structural violence is entrenched might also reproduce certain violence dynamics within the school environment itself as pointed out by recent studies [24, 25].

2) Relationships with significant support figures: Peer relationships that are mostly developed during the time spent in educational centers come first in the list of adolescents’ priorities [26, 27]. Adolescents need to be recognized by their peers, and the social support received from the latter is essential to strengthen their self-esteem [28]. Preadolescence and transition to secondary school are especially prone to bullying, due to the physical and psychological changes that occur during these periods [29, 2, 30]. Thus, when adolescents are perceived as different from others, whether because of their ethnicity, sex, socioeconomic status, the probabilities of being assaulted by their peers rise [31]. The same is true for psychological variables like depression [32], which may be one of the consequences of victimization, since violence, especially from peers, undermines self-esteem and also makes adolescents feel helpless and depressed [33, 34, 35, 32]. Additionally, psychosocial adjustment problems and low self-esteem sometimes make victims of abuse become aggressors [36, 37, 38]. Therefore, several studies consider victimization and bullying as part of a same phenomenon [39].

The consequences of bullying may be even more severe if there is a lack of socio-emotional support [40, 2, 41]. In this sense, the affective relationship that teachers establish with students are a key factor for school adaptation [42, 6, 43] and for other variables related to adolescent adjustment, such as social functioning (e.g., [44]), behavior problems (e.g., [45]), and academic achievement [46, 47, 48]. This is confirmed by a recent study conducted by Wang, Brinkworth and Eccles with 1400 youngster, showing that trust and positive affective relationships with teachers act as moderators in the relationship between parent-children conflicts, and depression and bad behavior in 13-to-18-year-old adolescents [49]. In this sense, the type of interactions that adolescents establish with significant people, depending on their developmental stage, appears to act as important protective or risk factors for victimization/bullying [3, 9].

### Present study

The constant changes that adolescents undergone with the onset of puberty makes this a time of special vulnerability, which contributes to the emergence of bullying [2]. From a social-ecological standpoint, it is estimated that violence relationships at a mesosystem level may influence the violence interactions that occur between peers during adolescence. In this regard, the structural violence of a context can turn affective relationships into risk factors rather than acting as protective factors [28].

Taking all this into consideration, the following hypotheses are proposed: (1) Perception of violence in the neighborhood where a school is located is expected to influence violence between peers within the educational center, as well as violence exercised by teachers in the classroom; (2) Violence relationships (i.e. between peers within the school and from teachers in the classroom) will be predictors of bullying behavior through victimization by peers; (3) Peer violence at school, violence exercised by teachers, and victimization will predict depression through loneliness; and (4) Differences will be found between early and late adolescence in the aforementioned variables.

Literature shows differences in aggressiveness and depression according to gender in adolescent population. Concretely, most studies report a higher prevalence of bullying cases in boys rather than in girls [50, 2]. Girls, on their part, would have greater levels of negative emotionality and depression [51, 52]. Therefore, gender was controlled for in the proposed structural equation model.

## Method

### Sample

Participants were part of an impact assessment study on school violence developed by the Ministry of Education of Peru, which sought to reduce the cases of violence in schools. Based on this criterion, Ministry of Education of Peru considered schools for this purpose and contacted directly with them’. In total, 21,416 13-to-17-year-old students in 70 schools from 19 different regions at a national level took part in the study (see Table 1). The mean age was 13.69 (SD = 0.71). As for the period of adolescence (early vs. late), most participants were in early adolescence. Thirty percent of the sample came from Lima province (i.e., Lima and Lima Callao), which has the greatest population of Peru (see Table 2).

**Table 1:** Demographic characteristics of participants (n = 21416).

Characteristic	n	%
Gender		
Male	11528	53.8%
Female	9888	46.2%
Stage of adolescence		
Early adolescence	14664	69.1%
Late adolescence	6559	30.9%

**Table 2:** Sample distributed by provinces.

Province	n	%
Amazonas	28	1%
Ancash	931	4.3%
Arequipa	604	2.8%
Ayacucho	248	1.2%
Cajamarca	102	05%
Cusco	58	0.3%
Huánuco	227	1.1%
Ica	3.143	14.7%
Junín	2.655	12.4%
La Libertad	1.799	8.4%
Lambayeque	1.897	8.9%
Lima	4.820	22.5%
Lima-Callao	1.586	7.4%
Pasco	233	1.1%
Piura	376	1.8%
Puno	804	3.8%
San Martín	570	2.7%
Tacna	1.128	5.3%
Tumbes	207	1%

### Procedure and ethics statement

The Ministry of Education of Peru directly contacted with all educational institutions. Parents and principals were informed of the scope and importance of the study, which lasted four months. All questionnaires were administered in the classroom in the presence of at least one member of the research team.

Ethical approval for this study was granted by the Ministry of Education of Peru (MINEDU). Parents were requested to sign an informed consent for the application of the questionnaire. To protect the privacy of the students, confidentiality of the questionnaires was guaranteed, and students consent was requested prior to their application. International ethical guidelines for studies with human subjects described in the Nuremberg Code and in the Declaration of Helsinki were applied.

### Measures

#### Reason for being bullied

Based on questions about the reasons for being bullied by other classmates. This scale was extracted from the Single School Well-being Questionnaire-CUBE (in Spanish, Cuestionario Único de Bienestar Escolar—CUBE) [53, 54]. This questionnaire was elaborated during the impact assessment carried out by the Ministry of Education of Peru to assess the development of socioemotional skills in educational institutions from Lima Metropolitan Area. This scale has 10 items that explore the reason for experiencing bullying (e.g. socioeconomic *status*). Each question was dichotomous, with *0* accounting for (“No), and *1* indicating (“Yes”). Kuder-Richardson reliability was .99.

#### Teacher’s aggressive behavior

The *Teacher Violence Scale* was based on questions about victimization extracted also from CUBE [53, 54]. This scale is composed by five items that enquire the student on violence situations directly (e.g. *A teacher threatened to hurt you or beat you*) or indirectly involving the teacher as the aggressor (e.g. *You saw a teacher insulting other student*), and whose range of response goes from 0 (“Never”) to 2 (“Two or more times”). Cronbach´s α for the studied sample was .90.

#### Bullying behavior

The *Bullying Behavior Scale* was based on the CUBE scale. This scale was composed by four items aimed at assessing the experience of the student as an aggressor (e.g. *I threatened to hurt or beat other student*). The response range goes from 0 (“Never”) to 2 (“Two or more times”). Cronbach´s α = .89.

#### Victimization

To measure this construct two scales were used: (i) The School Well-being Questionnaire and (ii) the inventory developed by Espelage and Holt [55]. This eight-item scale assesses the frequency of victimization suffered by the student considering the following categories: physical, verbal, exclusion, and cyberbullying (e.g. *One or more students insulted you*). The response range is from 0 (“Never”) to 2 (“Two or more times) Cronbach´s α was = .92.

#### Loneliness

Three-item was used to measure this construct, which is based on a brief version of the 20-item UCLA Loneliness Scale Test (e.g. *You feel isolated from others*). The responses for these questions range from 1 (“rarely or never”) to 4 (“Most or all the time”). The advantage of this shorter version is that its application is more practical and less expensive than that of the original scale. It is worth noticing that this version showed appropriate psychometric properties in previous studies [56, 57]. Cronbach´s α = .88.

#### Violence in school environment

Three-item self-constructed scale was used to identify vulnerability to violence in the school surroundings. The items are: (1) *There are places in the way to or back from school that I don’t like to go through because I’m afraid that somebody hurts me*; (2) *In my school many students are in gangs* and; (3) *Crime and violence in my neighborhood are affecting my school*, and whose responses range from 0 (“No”) to 4 (“always”). Cronbach´s α = .71.

#### School violence

Four items was used to assess this variable, based on the abbreviated version of the California School Climate and Safety Survey [58] (e.g. *The students of my school get into fights*), and with response categories ranging from 0 (“No”) to 4 (“always”). Cronbach´s α was .77

#### Depression

10-item *Depression Symptoms Test* developed by Bradley [59] was included. This test consists of a shorter version of the originally 20-item a CES-D [60]. An example item on the scale is: *I felt that everything I did was an effort*. Response options range from 1 (“rarely or never”) to 4 (“Most or all the time”). Cronbach´s α was .79.

All scales included in this study achieved the cut-off point of .70 proposed by [61]

### Statistical analysis

Descriptive statistics and correlations were calculated by means of the statistical program SPSS 22.0. The statistical package AMOS 18.0 was used for structural equation models, and multiple indirect effects were calculated using maximum likelihood estimation together with bias-corrected confidence interval bootstrap test. This procedure provides an average of the estimates obtained from bootstrap samples and their standard error. To verify the model fit, absolute and relative goodness-of-fit indices of the model were used, that is: χ^2^ indicators and the *χ*^*2*^*/gl* coefficient, Comparative Fit Index (CFI), Incremental Fit Index (IFI), Root Mean Square Error of Approximation (RMSEA) and Standardized Root Mean Square Residual (SRMR). A multi-group analysis was also run to verify differences between the early adolescence and late adolescence models.

## Results

### Descriptive analysis

#### Prevalence of exposure to violence

First, percentages were obtained through the responses to the Likert-type scales. The prevalence of violence in school environment, violence into school, as well as the presence of violent gangs were calculated taking as reference the response option corresponding to “sometimes” in the answers given to the scales measuring violence. In order to calculate the prevalence of violence suffered by teachers and peers, the answers that reported at least one aggression or abuse during the last month were considered.

Results show that 9,209 students (43%) felt unsafe when walking around the school surroundings. In addition, 12,721 (59.4%) adolescents reported usually seeing violent behaviors in school and 10,494 (49%) informed that youngsters from school belonged to violent gangs. Furthermore, 8,673 (40.5%) students reported having suffered abuse or insults from their peers at school. Finally, 4,237 adolescents (20.3%) stated having suffered or observed some type of violence from the teacher in the classroom. Table 3 summarizes the reasons for bullying behaviors according to the responses of the dichotomous scale.

**Table 3:** Prevalence of reasons for being bullied in male children (n = 11528) and female children (n = 9888).

	Male children		Female children		
Targets	n	%	N	%	χ2
a. Racial differences	975	8	717	7	6.17**
b. Geographical differences	514	4	310	3	20.44***
c. Sexual condition	603	5	302	3	55.12***
d. Physical appearance	2434	21	1780	18	20.34***
e. Presence of disabilities	293	3	128	1	38.54***
f. Religious beliefs	806	7	538	5	16.14***
g. Socioeconomic status	1192	10	736	7	43.44***
h. School grades (for both high and low scores)	1872	16	1437	15	5.22*
i. Without apparent reason	1917	17	1191	12	71.87***
j. Other reasons	2245	19	1734	18	6.32**

* p < .05

** p < .01

*** p < .001.

When comparing gender, men presented higher levels of aggressiveness (*M* = .44 vs. *M* = .28; *p <* .001), whereas women present higher levels of depression (*M* = 2.90 vs. *M* = 2.00; *p <* .001).

#### Correlations

Correlations were performed between all the study variables. As shown in Table 4, all correlations were significant and with values above .15.

**Table 4:** Correlations and Cronbach´s α between all the variables.

Variable	*1*.	*2*.	*3*.	*4*.	*5*.	*6*.	*7*.
1. Violence in the environment	—						
2. School violence	.36***	(.71)					
3. Teacher’s aggressive behavior	.26***	.36***	(.90)				
4. Loneliness	.24***	.20***	.15***	(.88)			
5.Victimization	.29***	.38***	.81***	.17***	(.92)		
6. Depression	.21***	.19***	.17***	.31***	.18***	(.79)	
7.Bullying behavior	.26***	.35***	.91***	.15***	.86***	.17***	(.89)

*** p < .001

### Measurement and structural indirect effects model

Firstly, to verify the fit of the variables into the model, Harman´s single factor test was conducted [62]. Two models were evaluated: (1) One latent factor model, in which all items were included; and (2) multiple latent factors model, in which the variables considered in this study were included. The results showed an adequate fit to the second model proposed. The model fit is as follows: *X²/df* = 1.66, *df* = 199; *p* < .001; CFI = .94; TLI = .93; SRMR = .04; RMSEA = .04.

**Fig 1:**
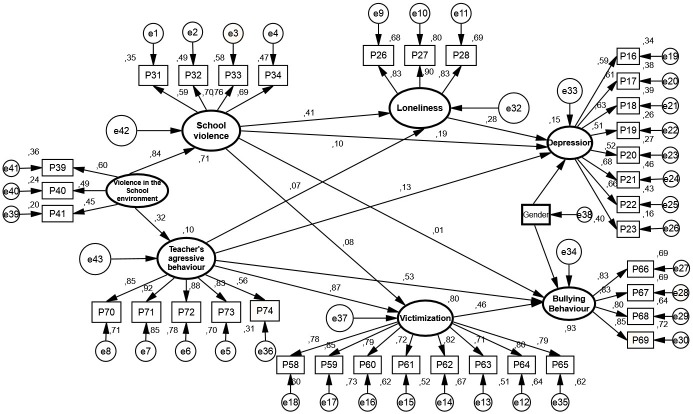
Multi-mediation structural equation model (SEM).

Subsequently, a first structural equation model was conducted to test the proposed hypotheses of this study. In this model, violent relationships in the school were considered as predictors of bullying behavior through victimization, and victimization by peers was taken as a predictor of depression through loneliness as suggested in hypothesis two and three. Nevertheless, it was observed that victimization by peers did not show a predictor effect on depression nor contribute to loneliness with more total variance explained. Therefore, a new model without that relationship was calculated. The results showed an adequate fit for the final model: *X²/df* = 1.66, *df* = 199; *p* < .001; CFI = .94; TLI = .93; SRMR = .04; RMSEA = .04 (Fig 1)

All direct effects of the model were significant (see Table 5). Regarding indirect effects with bias-correct confidence intervals, an indirect effect is observed in the relationship between school violence and depression through loneliness (*CI* = [.021, .134]); *p* = 0.01); and between school violence and bullying behavior through victimization (CI = [.029, .044]); *p* = 0.05). Furthermore, a significant indirect effect between teacher’s aggressive behavior and bullying behavior through victimization (CI = [.350, .459]); *p* = 0.05), and between teacher’s aggressive behavior and depression through loneliness (CI = [.013, .044]); *p* = 0.01) was identified. The variables included in the model explain 15% the variance of depression and 93% of the full model variance explained.

**Table 5:** Standardized direct effects of the model.

Dependent Variables		Independent Variables	Direct path estimate
School violence	<—	Violence in the school environment	.84***[.823, .886]
Teacher’s aggressive behavior	<—	Violence in the school environment	.31***[.291, .327]
Loneliness	<—	School violence	.41***[.378, .442]
Depression	<—	School violence	.09*[.065, .125]
Victimization	<—	School violence	.08* [.063, .090]
Bullying behavior	<—	School violence	.13**[.104, .150]
Loneliness	<—	Teacher’s aggressive behavior	.07*[.063, .090]
Victimization	<—	Teacher’s aggressive behavior	.87***[.854, .886]
Bullying behavior	<—	Teacher’s aggressive behavior	.63***[.567, .690]
Depression	<—	Teacher’s aggressive behavior	.17***[.128, .195]
Depression	<—	Loneliness	.28***[.254, .315]
Bullying behavior	<—	Victimization	.46***[.405, .528]

* p < .05

** p < .01

*** p < .001. Note: 95% intervals are in brackets

### Invariance across adolescence stage

**Fig 2:**
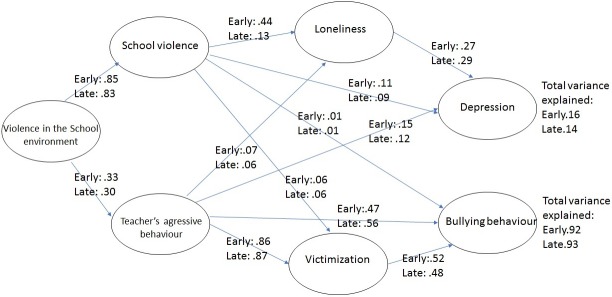
Comparison between early and late adolescence SEM models.

To calculate the invariance between the early and late adolescence models, a multi-group analysis was conducted. Significant differences were identified between the unconstrained and measurement weight models (*p* < .001). Specifically, slightly stronger relationships in path estimates were observed in the early adolescence model, although total variance explained in both models remain similar (Table 6; Fig 2). In the two models, indirect effects through victimization and loneliness were significant.

**Table 6:** Invariance between models of early and late adolescence.

Models	χ^2^	df	χ^2^/df	Δχ^2^	Δdf	CFI	TLI	SRMR	RMSEA
Model 1	14184	1162	-	-		.96	.96	.02	.02
Model 2	14305	1190	5.20	120.82*	28	.96	.96	.02	.02
Model 3	14353	1204	5.02	171.22*	43	.96	.96	.02	.02
Model 4	15470	1247	5.40	193.66*	50	.96	.96	.02	.02

* p < .001 Model 1: Unconstrained; Model 2: Measurement weights; Model 3: Structural covariances; Measurement residuals

## Discussion

First, descriptive data show that almost half of Peruvian youngsters perceives school’s environment as unsafe. In particular, 43% feel unsafe when walking down the streets surrounding school, and 49% reports that students from their school belong to violent gangs. This data is consistent with a high prevalence of peer victimization. Specifically, more than 40% of the sample informs having suffered from any type of physical or verbal violence, which widely exceeds data found by other studies reporting a prevalence of 20% (see review [2]). Likewise, almost 60% of students have observed any kind of violence in school, and more than 20% states having suffered or observed any kind of violence from the teacher in the classroom. These data are especially relevant, since they allow for an overview of violence exposure in countries like Peru, where violence is also structural in many of its regions.

Using a large sample of adolescents with different ages, this study confirms the great number of bullying cases observed in recent studies on adolescent population in Peru (see review [22]). Descriptive results also show a greater prevalence of all victimization targets in boys, compared to girls. These data are consistent with most studies that concluded that bullying behavior is more common in boys, who thus suffer more victimization (e.g. [2, 63]). In gender, the most common target is direct-verbal behavior, specifically teasing due to physical appearance, and simply to provoke conflict. Other target with high presence is comparison because of academic grades. Both boys and girls also report a high prevalence of other non-typified causes that might be more related to indirect targets, e.g. spreading rumors, cyberbullying, etc., according to Wang’s classification [64]. Regarding differences in the prevalence of bullying behavior and depression by gender, results are in line with other previous studies, with a higher prevalence of depression in girls and of bullying behavior in boys.

According to the first hypothesis, results show that violence relationships in the neighborhood enhance violence relationships between peers in school, as well as between teachers and students. These results are extremely relevant, as they indicate the existence of a clear relationship between violence perceived in the school environment and the influence of the adolescent’s main support figures within educational centers. Recent studies already demonstrated the negative relationship between violence in the neighborhood and socio-emotional and respect climate in school [65, 25]. However, some contradictions concerning the influence of school environment on bullying behavior still exist in those results [2]. The results of this study show the indirect influence of violence perceived in the school environment on bullying behavior through violence exercised by support figures (peers and teachers).

In this sense, results confirm the existence of an indirect effect on the relationship between violent interactions (with peers in school and teachers) and bullying behavior through peer victimization, thereby confirming the second hypothesis. It must be noted that violence from teachers explains better the emergence of aggressive behavior in the adolescent than exposure to the perception of peer violence at school. However, peer victimization is also a predictor of bullying behavior. Former studies characterized bullying victims with a profile different from that of youngsters that are either victims or aggressors (e.g., [66, 67, 68]). Accordingly, results show that being a victim of peers strengthens the relationship between violence from the teacher in the classroom and the adoption of aggressive behaviors. Therefore, if all interactions with significant supports are based on violence, the imitation of these behaviors could intensify. Results show that violence exercised by peers and teachers in the school environment explains more than 90% of the total variance explained for bullying behavior.

As for the third hypothesis, both violence from peers in school and violence from teachers in the classroom are two clear predictors of depression through loneliness. Nevertheless, this is not true for peer victimization, since it neither shows a predictor effect on loneliness nor on depression after being included into the model. In addition, peer victimization does not contribute to a greater explained variance for depression. Consequently, the third hypothesis is only partly confirmed. Results show that violence relationships between the peer groups in school lead to a perception of loneliness in adolescents that, in turn, generates greater depression levels. Therefore, and as pointed out in several studies, social support is a fundamental factor to prevent depression [69, 70]. The same applies to the effect of violence from teachers over depression, in which an indirect effect is also seen through loneliness. In this regard, it must be highlighted the role of the interaction between adolescent students and teachers in the classroom in severe psychological problems such as depression. So, if i*nteraction with teachers results to be a positive relationship based on confidence, this may generate an important cushion effect when symptoms appear [49] in this stage marked by developmental changes [71].

Finally, differences between the early and late adolescence models confirm the fourth hypothesis. Overall, predictive relationships with victimizations and loneliness and other dependent variables are less strong in late adolescence. In this sense, the literature points out that early adolescence is the most complex stage, since adolescents undergo physical and psychological changes and transit from primary to middle school [72, 73]. Therefore, weight should be given to these first stages of transition to adolescence, since they are crucial to prevent bullying situations from entrenching.

Despite the differences observed between early and late adolescence models, the indirect effects between aggressiveness relationships at home and from teachers, and aggressive behavior and depression through mediators remain the same. The model, thus, explains both adolescence stages, and the total variance explained by the different variables of the model is almost in the same values for both depression and bullying behavior. This reinforces the need of continuing to adopt effective strategies for preventing bullying, which imply improving affective relationships and strengthening support figures during adolescence.

The sample used in this study comprises early and late adolescence, which allowed us to identify differences between these two developmental stages. However, there is an increasing need for longitudinal studies that enable the observation of abiding violence situations in cases of bullying. Consequently, we suggest that future studies address the period from late childhood to the end of adolescence in order to analyze changes in relationships at a mesosystem level, as well as the influence of these changes on the perpetuation of violence. In future research, it would be interesting to assess this phenomenon taking into account the different agents involved in the child’s ecological setting and contrast differences by means of multi-level analysis.

## Conclusions

First, the high levels of exposure to violence reported by Peruvian adolescents at both neighborhoods where schools are located and within educational centers are noteworthy. An increasing awareness of this issue has been seen in public institutions over the past few years, but interventions are still scarce and insufficient [74]. The prevalence of structural violence in school environments calls for specific measures that allow for preventing violence in a comprehensive way and from an ecological approach. To this end, a strong coordination among the different services and agents at a community level is necessary for taking ecological preventive actions in which school, family and community work together. Results also show that relationships based on violence from significant figures for the adolescent, such as peers and teachers, contribute to the emergence of aggressive behavior and depressive symptoms. In this sense, teachers are the significant adults that should act as positive and stable socio-emotional supports inside schools, and assist in preventing peer conflict during adolescence. The aforementioned requires teachers to be specifically trained to deal with violence situations and to establish effective strategies to improve classroom climate and, in turn, enhance students’ academic performance [75, 76]. Otherwise, the prevalence of bullying situations will increase, thus causing the reproduction of violent behavior patterns and the ultimate entrenchment of violence.
